# Total and Partial Laser Arytenoidectomy for Bilateral Vocal Fold Paralysis

**DOI:** 10.1155/2016/3601612

**Published:** 2016-10-17

**Authors:** Taner Yılmaz, Ozan Muzaffer Altuntaş, Nilda Süslü, Gamze Atay, Serdar Özer, Oğuz Kuşçu, Tevfik Sözen

**Affiliations:** ^1^Department of Otolaryngology-Head and Neck Surgery, Hacettepe University Faculty of Medicine, Ankara, Turkey; ^2^Department of Otolaryngology-Head and Neck Surgery, Ankara Numune Research and Training Hospital, Ankara, Turkey

## Abstract

*Introduction*. Treatment for bilateral vocal fold paralysis (BVFP) has evolved from external irreversible procedures to endolaryngeal laser surgery with greater focus on anatomic and functional preservation. Since the introduction of endolaryngeal laser arytenoidectomy, certain modifications have been described, such as partial resection procedures and mucosa sparing techniques as opposed to total arytenoidectomy.* Discussion*. The primary outcome measure in studies on BVFP treatment using total or partial arytenoidectomy is avoidance of tracheotomy or decannulation and reported success ranges between 90 and 100% in this regard. Phonation is invariably affected and arytenoidectomy worsens both aerodynamic and acoustic vocal properties. Recent reports indicate that partial and total arytenoidectomies have similar outcome in respect to phonation and swallowing. We use CO_2_ laser assisted partial arytenoidectomy with a posteromedially based mucosal flap for primary cases and reserve total arytenoidectomy for revision. Lateral suturing of preserved mucosa provides tension on the vocal fold leading to better voice and leaves no raw surgical field to unpredictable scarring or granulation.* Conclusion*. Arytenoidectomy as a permanent static procedure remains a traditional yet sound choice in the treatment of BVFP. Laser dissection provides a precise dissection in a narrow surgical field and the possibility to perform partial arytenoidectomy.

## 1. Introduction

From Theodore Maiman's introduction of the ruby laser [1] to the scientific literature to the advent of carbon dioxide (CO_2_) laser surgery, the journey of lasers in applied medicine and otolaryngology has encompassed more than six decades. The pioneering work of Geza Jako, who not only invented numerous cold steel microlaryngeal instruments but also conducted the first CO_2_ laser laryngosurgery on canine subjects [2], gave the field of laryngology a potent tool to surpass its unique challenges. In accord with the requirements of endoscopic microsurgery, laser provides precise dissection, superior exposure due to hemostasis, and the ability to work at large distances with less tissue manipulation.

Larynx tissue has ideal properties for CO_2_ laser use: laryngeal mucosa absorbs the infrared (10,600 nm) wavelength quite well owing to its high water content and high focus provides limited penetration with minimal collateral thermal damage. Considering these advantages, laser laryngosurgery was first utilized in early laryngeal cancer by Strong [3] in 1975 who reported precision for surgical margins and satisfactory postoperative healing after vocal fold resection in 11 cases. Soon, CO_2_ laser use was expanded to various benign conditions of the larynx, such as recurrent respiratory papillomatosis [4], laryngeal stenosis [5], and the surgical management of bilateral vocal fold paralysis [6] (BVFP).

BVFP is a catastrophic complication of neck surgery and its treatment has evolved markedly since Jackson proposed external ventriculocordectomy as an alternative to tracheotomy in 1922 [7]. Later approaches included Woodman's external total arytenoidectomy [8] and Thornell's eponymous endoscopic arytenoidectomy [9] in an attempt to widen the posterior glottic airway. Thornell operation is still widely utilized as an effective method to prevent tracheotomy or achieve decannulation, albeit with certain modifications. After the advent of CO_2_ laser laryngosurgery, Ossoff et al. [6, 10] applied laser dissection to vaporize the arytenoid and treated a total of 28 patients that were all successfully decannulated in a three-year span. Further refinement of the technique was done by Crumley [11] who proposed that endolaryngeal laser medial arytenoidectomy (i.e., partial arytenoidectomy with resection of the medial arytenoid body and preservation of the vocal fold attachment) provided comparable success with less vocal distortion for cases “seeking a small increment” in airway caliber.

In this review, a comprehensive look into endolaryngeal arytenoidectomy with the aid of CO_2_ laser will be provided. The authors aim to detail the current operative technique and discuss the merits of the procedure, potential success rate, and possible complications in the light of current literature.

## 2. Laser Arytenoidectomy Procedure

### 2.1. Preoperative Considerations

The vast majority of BVFP cases are due to iatrogenic recurrent laryngeal nerve palsy, with 48.6% of patients having undergone thyroidectomy [12]. Vocal fold immobility after surgical trauma may not be permanent if the nerve has not been transected and a follow-up period of 6 months to 1 year is advocated with tracheotomy or suture lateralization as a temporizing measure if necessary. If BVFP is deemed permanent, the patient should be informed on arytenoidectomy as an operative option and its probable consequences. It should be thoroughly emphasized that, after irreversible static laryngeal procedures, a gain in airway caliber will almost certainly translate into a decline in voice quality. Additional morbidity that may impact pulmonary reserve should be addressed before the operation (chronic obstructive pulmonary disease, previous lung resection, etc.). Needless to say, appropriate informed consent should be obtained as airway surgery always entails a significant operative risk. Patients without tracheotomy should be informed about possibility of postoperative airway obstruction and tracheotomy and appropriate informed consent about tracheotomy should be obtained.

### 2.2. Total Arytenoidectomy Operative Technique

The anesthesiology team should be aware of the possibility of a difficult intubation beforehand if the patient does not have a tracheotomy and all precautions including those pertaining to emergency surgical access to the airway should be taken. A small caliber endotracheal tube (6.0 mm or less if possible) is used to improve endoscopic visualization. Laser compatible, shielded endotracheal tubes provide an additional layer of protection against airway fire and should be preferred if present.

The authors prefer a modified Kleinsasser direct laryngoscopy blade built and patented by the senior author [13] (TY). A groove in the lingual surface of the laryngoscope retracts the endotracheal tube upward and to the left, which facilitates access to the posterior glottis and the right arytenoid. Damp gauze may be draped over the patient's face to prevent accidental laser burns and wet neurosurgical pledgets may be placed around the endotracheal tube to shield against laser penetration. While the laser is active, ventilation is switched to room air as 100% oxygen poses an additional fire hazard.

**Figure 1:**
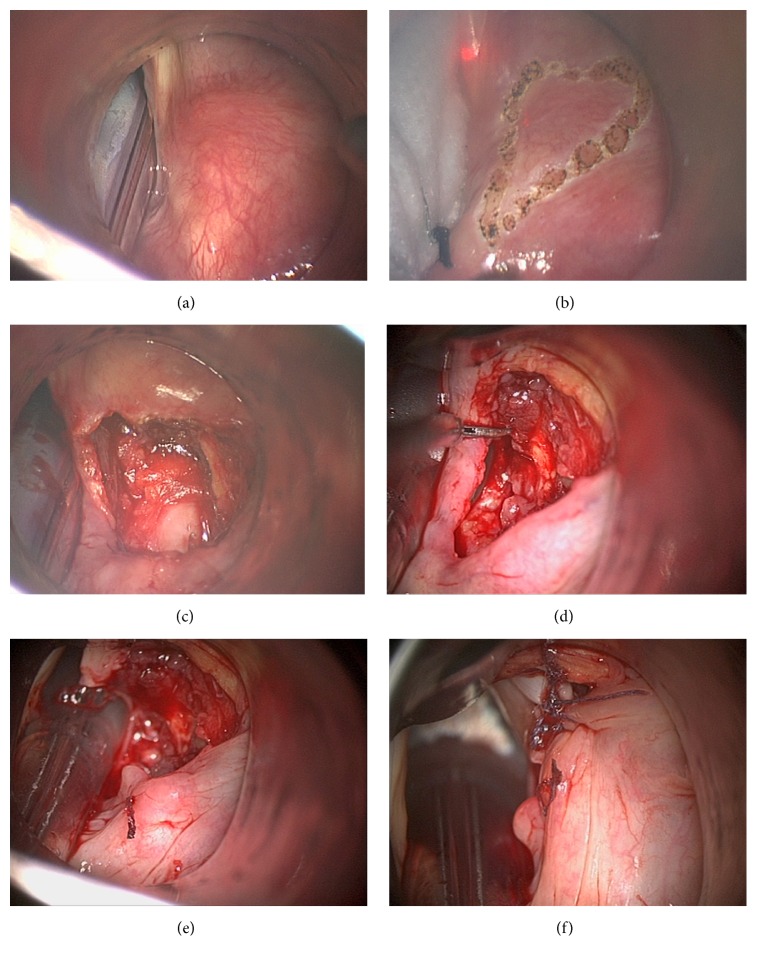
(a) Right arytenoid cartilage is visualized with regular laryngoscope; intubation tube is within glottis. (b) Anteriorly based triangular incision was marked with CO_2_ laser spots on right arytenoid. (c) After removal of right arytenoid cartilage, cricoarytenoid joint surface is visualized; mucosa medial to arytenoid is preserved to be used as a flap. (d) Mucosa medial to arytenoid was preserved and is about to be cut right behind membranous vocal fold to be used as a flap later. (e) Posteromedially based advancement flap is sutured posterolaterally. (f) Membranous vocal fold was sutured posterolaterally; glottis is enlarged.

Laryngoscopy should commence with assessment of patient's anatomy and palpation of the cricoarytenoid (CA) joint to rule out posterior glottic stenosis. The incision is begun with the CO_2_ laser in cutting mode at 5 watts and continuous mode on the arytenoid mucosa as a triangular flap with its corner towards the apex of arytenoid cartilage which may then be discarded. Further dissection will expose the arytenoid apex and the vocal process of the arytenoid cartilage. From now on the operation continues with cold instruments. The vocal ligament is sharply freed of its insertion. Posterior lamina of the cricoid cartilage is encountered in the deepest extent of the incision below the arytenoid cartilage and the CA joint capsule is sharply divided. Arytenoid cartilage is removed totally with the aid of conventional cold steel instruments. Care must be utilized not to damage cricoid cartilage; otherwise subglottic stenosis may ensue. During dissection of arytenoid, mucosa medial to arytenoid is carefully preserved to be later used as an advancement flap. On the mucosa medial to arytenoid a vertical mucosal cut is made right behind the membranous cord towards subglottis. This maneuver produces posteroinferiorly based mucosal advancement flap which will be sutured to the previous place of muscular process of arytenoid, thus enlarging the posterior glottis. One or two 5/0 vicryl sutures are placed endoscopically between the anterior edge of mucosal flap and surgical bed around the previous place of muscular process of arytenoid. The free membranous vocal cord is sutured posterolaterally to the aryepiglottic fold right lateral to the previous advancement flap suture with three or four sutures of 5-0 vicryl, thus tensing and lateralizing vocal fold (Figures 1(a)–1(f)). This maneuver both enlarges glottis and preserves voice. Both of these sutures enable the surgeon to cover open surgical areas with mucosa, thus decreasing the risk of granulation tissue formation. Posterior commissure mucosa should not be traumatized to prevent posterior glottis stenosis. This modification of the classical arytenoidectomy technique prevents unpredictable healing by scar contracture and preserves the tension on the vocal fold.

Final airway size can be assessed in the course of the procedure. Extubation should be uneventful, if the posterior glottis is satisfactorily widened.

### 2.3. Partial Arytenoidectomy Operative Technique

**Figure 2:**
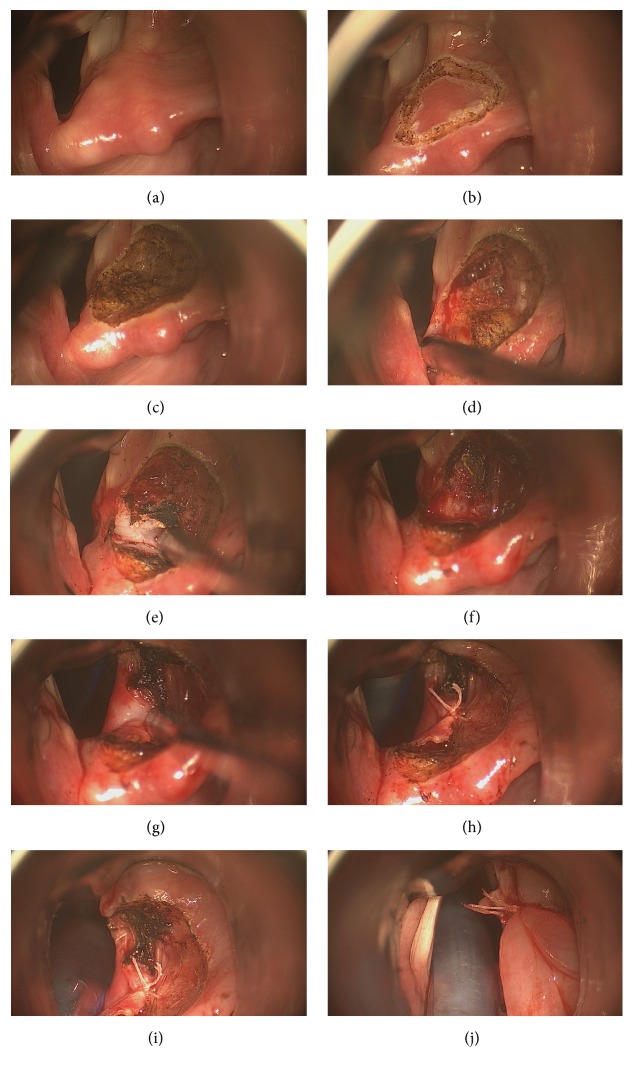
(a) Right arytenoid cartilage is visualized with modified laryngoscope; intubation tube is elevated with this laryngoscope out of surgical field, thus enlarging field of vision. (b) Anteriorly based triangular incision was marked with CO_2_ laser spots on right arytenoid. (c) Mucosa covering arytenoid was removed revealing superior surface of cartilage. (d) Anterior half of arytenoid was dissected; mucosa medial to arytenoid was preserved. (e) Anterior half of arytenoid was cut with CO_2_ laser transversely and is about to be removed. (f) Anterior half of arytenoid was removed. Mucosa medial to arytenoid was preserved. (g) Posteromedially based advancement flap was outlined and shown. (h) Posteromedially based advancement flap was sutured posterolaterally. (i) After posteromedially based advancement flap is sutured posterolaterally, membranous vocal fold is about to be sutured posterolaterally. (j) Membranous vocal fold was sutured posterolaterally; glottis is enlarged.

Surgical procedure is similar to total arytenoidectomy until arytenoid cartilage is freed from medial mucosa and lateral soft tissue attachments towards muscular process. The muscular process and the posterior wall of the arytenoid are not dissected. For partial arytenoidectomy, laser incision is made transversely in the middle of the body of arytenoid down to the cricoarytenoid joint; after the incision is completed, vocal process and anterior half of body of arytenoid are removed with cup forceps piecemeally. Posterior half of body and muscular process are preserved. Interarytenoid, thyroarytenoid, and lateral cricoarytenoid muscle attachments are severed, preserving posterior cricoarytenoid muscle attachment to the posterior face of muscular process. Similarly, mucosa medial to arytenoid is preserved; it is cut right behind the membranous vocal fold. This cut produces posteriorly and medially based mucosal flap. This posteriorly and medially based mucosal advancement flap is sutured posterolaterally over the remnant body of arytenoid to the lateral wall of surgical wound enlarging posterior glottis and decreasing the size of the open surgical wound. Vocal fold lateralization is done with endoscopic microsuture by suturing the membranous vocal fold lateral to the remnant body of arytenoid, thus covering all open wounds. This microsuture also tenses the already flaccid membranous vocal fold, thus preserving voice (Figures 2(a)–2(j)).

### 2.4. Postoperative Care

Authors do not routinely use antibiotics, analgesics, or antireflux medications postoperatively in all patients undergoing laser laryngosurgery. 250 mg methyl prednisolone is infused intravenously towards the end of the operation. The patient should be closely monitored for airway edema and impending obstruction, if a tracheotomy is not present. When signs of airway obstruction develop, another 250 mg methyl prednisolone is given intravenously; if airway obstruction continues, tracheotomy should be performed. Among 150 personal cases of total and partial arytenoidectomies without tracheotomy, only 4 patients (2.67%) required postoperative tracheotomy. Normal feeding is resumed six hours after surgery. Decannulation is usually achieved at the end of first postoperative month after early postoperative edema subsides. Antireflux medication may be prescribed to reduce inflammation in the healing posterior glottis.

## 3. Discussion

Treatment for BVFP includes static and dynamic surgical alternatives, none of which perfectly restore the form and function of the human larynx. As a very sophisticated neuromuscular apparatus that periodically permits inspiration, guards the airway during deglutition, and provides vocalization over an impressive range of loudness and frequency at the same time, any major intrusion to its structure is determined to somewhat impair one or more of these capacities.

There is a wide array of studies on endolaryngeal laser (or laser assisted) arytenoidectomy in the English literature with varying methodology, while the primary outcome measure after laser arytenoidectomy remains respiratory function as reflected by the avoidance of tracheotomy or the possibility of decannulation. The parameters investigated may be categorized as success with regard to airway compromise, voice outcome, and short- or long-term complications such as airway edema, granuloma formation at the surgical field, or aspiration.

Endoscopic laser arytenoidectomy was described on a total of 28 patients by Ossoff et al. [10] as a procedure comprising total vaporization of the arytenoid and its mucosa, leaving the surgical bed to healing by secondary intention and scar contracture. It has been postulated that this approach leaves the precise amount of glottic widening to chance, leading to granulomas on the surgical site due to the mucosal gap, and facilitates functional loss by leaving the remaining vocal fold devoid of tension and posterior glottis susceptible to aspiration. In accordance with these criticisms, several modifications were proposed to alleviate probable complications of the procedure.

Rontal and Rontal advocated the use of endolaryngeally sutured microtrapdoor flaps [14] and selective tenotomy of the interarytenoid and thyroarytenoid muscles [15] which permitted a certain extent of conservation for the arytenoid cartilage. Remacle et al. [16] proposed subtotal resection of the arytenoid, leaving a posterior shell of bone and the interarytenoid cleft intact. It is stressed that this modification leads to less aspiration and avoids posterior glottic scarring in their original report. Crumley's endoscopic laser medial arytenoidectomy (ELMA) introduced further conservation by submucosally resecting the medial part of the arytenoid [11] to achieve a 1-2 mm posterior glottic chink. While it is credited as being the least invasive approach to arytenoid, this operation provides very small increment of glottic caliber and is frequently required bilaterally.

A prospective cohort of 50 patients with BVFP by the senior author [17] is currently one of the largest cohorts in the literature and reports 90% success using CO_2_ laser assisted total arytenoidectomy with a posteromedially based mucosal advancement microflap. Predictably, postoperative voice parameters such as Voice Handicap Index-30 (VHI-30), maximum phonation time (MPT), absolute jitter, and shimmer percentage have worsened significantly in this series, yet swallowing parameters were not altered by functional outcome swallowing scale (FOSS). Three out of 40 patients that did not have a tracheotomy preoperatively required one temporarily due to airway edema after surgery. No postoperative granulomas were observed presumably because of the mucosal restoration provided by the microflap advancement technique.

A continuation of Remacle's subtotal arytenoidectomy series is reported in a study by Plouin-Gaudon et al. [18], in which 100% success was obtained with regard to respiratory function in a cohort of 69 patients. In a subset of patients, postoperative voice analyses were undertaken and revealed worse MPT. The authors report some aspiration essentially with liquids that spontaneously resolved in days to weeks after surgery. These findings suggest that subtotal arytenoidectomy distorts the voice no less than other approaches and while persistent aspiration is not noted, early postoperative days require some caution with regard to the patient's diet.

A newer prospective cohort by Gorphe et al. [19] evaluated 20 patients after ELMA as described by Crumley. While all patients were successfully treated with respect to respiratory function, 18 required bilateral ELMA to establish sufficient airway. Postoperative voice parameters were available in eight patients and demonstrated worsened jitter and shimmer yet unchanged MPT. Authors interestingly found an improvement in the emotional component of the VHI-30 questionnaire, while physical and functional components were not significantly altered. It seems possible by these findings that a patient in need of a marginal increase in airway caliber may benefit from this procedure with less vocal distortion.

While frequently there is concern towards marked vocal distortion and risk of aspiration after total arytenoidectomy, these fears may not be entirely justified. In a trial of 20 patients with BVFP randomized between total and partial (resecting the vocal process and anterior body) arytenoidectomies, Yilmaz et al. [20] found that neither approach had different results regarding success rate, VHI-30 questionnaire scores, aerodynamic, acoustic analysis, or FOSS. The single distinction found was the length of the procedure: partial arytenoidectomy took 10 minutes longer to perform on average.

Some authors believe that an additional measure such as posterior true or false cordotomy may be necessary in addition to arytenoidectomy to guarantee improvement in airway caliber. Maurizi et al. [21] performed subtotal arytenoidectomy and posterior true and false cordotomy in 39 cases with 100% success. Vocal analyses were distorted with marked aperiodicity noted in the voice signal and MPT was prolonged. Misiolek et al. [22] reported the long-term follow-up results of such an approach (arytenoidectomy and posterior cordectomy) in a series of 30 patients, 100% of which remain decannulated. Sadly, no objective vocal analysis was performed in this study.

We do not remove any part of the membranous true vocal fold or vestibular fold. If a part of true or false vocal fold is removed, this does not increase postoperative airway size. If a part of true vocal fold is removed, this will certainly worsen voice. Our surgical technique is a mucosa preserving technique; we do not burn mucosa and underlying arytenoid cartilage down to the cricoarytenoid joint. Total destruction of mucosa with laser will lead to extensive and uncontrolled scar tissue formation and may result in posterior glottis stenosis. This may also cause chondritis of cricoid by decreasing the vascularity of laser-treated mucosa. The use of mucosa as flaps not only will enlarge posterior glottis but also will decrease the risk of granulation tissue formation. As we lateralize membranous vocal fold with microsutures, we suture it posterolaterally; if a more lateral position is chosen for suture placement, the glottis will be larger in size; however, the voice outcome will be worse. Thus, for a primary case, a posterolateral suture position is ideal, while a more lateral suture position is better for a revision case, where patient needs more airway. Partial arytenoidectomy has the advantage over total arytenoidectomy of preserving the height of the arytenoid cartilage; this height may decrease the risk of aspiration by preventing free flow of piriform sinus contents into the larynx. Even though research shows no increased risk of aspiration after total arytenoidectomy compared to partial arytenoidectomy, it is sound to be on the more conservative side and perform partial arytenoidectomy for primary cases and reserve total arytenoidectomy for revisions. Since 2012, our treatment policy for bilateral vocal fold paralysis has been to perform partial arytenoidectomy for primary cases and reserve total arytenoidectomy for revisions of partial arytenoidectomy.

Success rate reported in the pediatric patients is 66–77% and markedly lower compared to adults [23, 24]. This fact is most probably due to the higher incidence of posterior glottic stenosis, subglottic stenosis, and other comorbid pathologies of the airway in the pediatric age group. Complications also seem to occur with greater frequency in these patients: a series by Aubry et al. [23] describes five cases of significant dysphonia and two cases with persistent aspiration among 17 operations.

Possible complications after laser arytenoidectomy include scar retraction leading to secondary glottic stenosis, cricoid cartilage chondritis, and granuloma formation in the surgical site in addition to the obvious risks of vocal deterioration and aspiration. Misiolek et al. [25] found that excised postoperative granulomas contained young granulation tissue with inflammation in histopathology. Antireflux medication is advised to reduce inflammatory effect of laryngopharyngeal reflux on the healing mucosa after arytenoidectomy. To the best of the authors' knowledge, there is no report of chondritis after laser arytenoidectomy in the current literature and the risk may be negated by the use of postoperative antibiotics.

## 4. Conclusion

Arytenoidectomy as a permanent static procedure remains a traditional yet sound choice in the treatment of BVFP due to its dependable rate of success and acceptable functional outcome. Laser dissection provides a hands-off, bloodless, precise dissection in a narrow surgical field. Ease of application and the possibility of partial arytenoidectomy procedures are further advantages.
